# Some Points in Artificial Immunity

**Published:** 1919-08-09

**Authors:** 


### DISEASE AND THE LABORATORY.

### II. Some Points in Artificial Immunity.

Throughout the past five years unexampled opportunity has been given to the medical departments of the Services for investigation of innumerable prevention problems. The masses of men under their control, the mobilisation of every research organisation in the country, every laboratory, and every medical unit for national service, has produced a unique phase in the history of both disease and the stresses to which human life can be subjected. At present, however, owing probably to the enormous amount of material on hand and the relatively short time since cessation of active hostilities, official announcements of progress, though clear and excellent individually, have been somewhat erratic in the date and form of their publication. The lay Press has even more erratically made selection from these separated components of a great national work, with the result that the public are frequently inclined to view the essentials of our new views on disease prevention and kindred problems in a somewhat misleading perspective. Particularly does this apply to infectious diseases—namely, those directly due to micro-organisms, and attention is here drawn to a few of the fundamental ideas and discoveries which have governed the recent highly successful work of Service laboratory organisations. Apart from general measures, artificial immunity has been a key-note of the times.

### The Study of Immunity.

In a number of diseases an attack is known to leave the sufferer relatively immune to subsequent invasions of the same infection; but even in these instances the object of the immunologist is to discover the means by which a man can be made immune *without* being subjected to the dangerous and disagreeable experience of a previous infection. Artificial immunity is best described as active, passive, or a combination of both. In active immunity the body produces its own immune substance in response to a stimulus artificially applied, and this is instanced typically by the inoculations with an emulsion of killed typhoid and paratyphoid bacilli containing the toxins of the organisms. The effect of the toxin on the body is an over-production of antitoxin, and, theoretically, it would seem possible to produce in this way an active immunity against any known organism; but practically it is not so. With the enteric fevers effects have been most striking, and as a prophylactic measure T.A.B. vaccine (that is a vaccine protecting against typhoid, paratyphoid A, and paratyphoid B) has no equal; but, nevertheless, it should be realised that the protection is *not* absolute, and also that we do not yet know the length of time protection lasts, and hence the intervals at which inoculation should be repeated. Other less successful instances occur with pneumonia, bacillary dysentery, local and general sepsis, etc. For example, the Americans reduced the incidence of pneumonia in some of their camps by 50 per cent. during the recent influenza outbreaks by the use of polyvalent killed vaccines of organisms common in pneumonias; similar inoculation against dysentery was relatively successful, and a certain degree of protection can be given against a host of common inflammatory organisms; but there are both limitations and objections to an indiscriminate faith in the value of these agents, especially the "stock" varieties.

One great drawback to active immunisation thus accomplished is the length of time required to develop the immunity. Actually, a period of hyper-susceptibility follows inoculation—the negative phase—and it is about ten days before the required excess of antitoxin is developed. Thus arises a definite risk if inoculation be made *during* epidemics, and a bar to the practice of giving, in *treatment* of acute infections, doses of vaccine sufficiently high to give a reasonable antitoxin production.

Stock and autogenous vaccines are now readily obtainable from a number of trade institutions, which willingly supply them without any person connected with the laboratory hearing more than the scantiest details of the patient's condition, and against relying on the indiscriminate use of these inoculations both the public and the profession must be more than careful. On the whole, considering the situation as it stands for the moment, vaccines and pure active immunity, except under the personal control of an expert and in certain definitely established chronic conditions, must be viewed as unsuitable for *treatment* of infectious disease, but most valuable in *prophylaxis*.

### Passive Immunity

For treatment, therefore, resort has to be made to passive immunity, accomplished by the introduction into the patient of immune serum produced by active immunisation of another animal. In this way an immediate immunity is conferred, but very temporary in nature, as neutralisation is strictly quantitative, and repeated injections must be made if the infection be not at once overcome. The extensive use of tetanus and diphtheria antitoxin is too well known to need description here. The only point, and one requiring the greatest emphasis, is that administration must be made at the earliest possible moment—in fact, whenever suspicion of possible infection arises—without waiting for any bacteriological confirmation. Lack of appreciation

The previous article appeared on July 26, p. 425.

**Disease and the Laboratory—(*continued*).**

of the time factor in all serum treatment will completely spoil the efficiency of the method.

To refer to some more recently produced sera, it may be explained that in the case of cerebro-spinal meningitis two new ideas have been practically applied. The usual intrathecal administration has of late been widely supplemented by intravenous methods. Investigations in the last two years suggest that acute C.S.M. infection tends to be general from the onset, and use of the double route has led to a very definite reduction in mortality. Still greater improvement will undoubtedly follow, for the meningococci have been divided into four distinct immunological types, for each of which an antiserum is now on the market; and it is worth noting very satisfactory results are already being obtained by immediately injecting, on the first suspicions of meningitis, full doses of polyvalent serum by the double route, sending, meanwhile, a specimen of C.S.F. and a naso-pharyngeal swab to the laboratory. Typing of the organism can be done in thirty-six hours, and from the end of that time treatment can be continued with antiserum for the correct type only.

### Other Sub-Species.

There are several further examples of sub-species among micro-organisms, each producing distinct toxins, notably those causing septic complications, or pneumonia, and the gram-positive cocci. Frequently not only are these sub-species all prevalent during any one local outbreak, but also there are distinct species proper to different countries. Clinically, the manifestations may be identical, but immunologically these germs differ very greatly. Success has, however, been obtained, mostly on the lines described for the meningococcus; polyvalent serum are at once exhibited, and continued until the correct type or autogenous product can be given.

### Anaphylaxis.

No description of serum therapy would be complete, however, without some reference to the phenomena of anaphylaxis. It is well known that symptoms of hyper-sensitiveness may appear on serum administration, whether the dose be an initial or later one, and that they are far more likely to occur when the intravenous route is utilised. What is, perhaps, not so well appreciated, and is certainly not generally applied at home, is that these phenomena can be largely avoided by slow administration, use of diluted sera, or preliminary desensitisation of the patient by a combination of these means. In any case the liability to anaphylaxis can generally be demonstrated by injection of a minute amount of serum under the skin, any local reaction being noted; but in practice anaphylaxis from these causes rarely results in death, in spite of often very alarming symptoms.

Unfortunately, to carry out such work with full efficiency not only more extensive time-saving organisation, but also a large amount of skilled laboratory technique, will be necessary throughout the country. In the Services we had good approximation to these, particularly in the closing years of the war, and it will be the task of the Ministry of Health to establish a condition at home progressing as rapidly as possible towards a similar centralisation. The secret lies, perhaps, in the degree of power which our health authorities are allowed to wield, the support they receive from the profession, and, on which these both depend, the forcefulness and wisdom with which the Ministry applies the experience the war has provided.

